# Encysted Vesical Calculi

**Published:** 1899-05-20

**Authors:** 


### ENCYSTED VESICAL CALCULI.

At the last ordinary meeting of the Medical Society Mr. Bruce Clarke read a paper giving an account of twenty-seven cases of encysted vesical calculi. He pointed out that in these cases, where the stone was fixed to the bladder wall, the usual symptoms of stone in the bladder were absent. Hæmaturia and pain rarely occurred, and cystitis was usually the only symptom, the stone often not being detected until the bladder was opened. The exact position of the stone in such cases varied considerably. Some were embedded in the prostate, some lay loose in cysts in other parts of the bladder, but usually near the base. Most of these cases occurred in individuals between sixty and seventy years of age, and the lesson taught by a study of their symptoms was the necessity for exploring doubtful cases of cystitis which did not yield to ordinary methods of treatment; and in regard to this exploration it was to be borne in mind that, although the cystoscope was sometimes of use, the stone was in many cases so completely embedded that it could hardly be seen or felt, even when the bladder was opened, so that a very complete exploration was necessary before it could be shown that a persisting cystitis was not due to an encysted calculus.

